# A Treat-to-Target approach in hereditary angioedema: expert consensus from a European committee

**DOI:** 10.3389/fimmu.2026.1773279

**Published:** 2026-02-12

**Authors:** Mauro Cancian, Teresa Caballero, Isabelle Boccon-Gibod, Thomas Buttgereit, Danny Cohn, Efrem Eren, Sorena Kiani-Alikhan, David Launay, Johanna Mandelin, Andrea Zanichelli, Henriette Farkas

**Affiliations:** 1 Department of Systems Medicine, University Hospital of Padua, Padua, Italy; 2 Department of Allergy, Hospital Universitario La Paz, Hospital La Paz Health Research Institute (IdiPAZ), Center for Biomedical Network Research on Rare Diseases (CIBERER) (U754), Madrid, Spain; 3 Department of Internal Medicine, National Reference Center for Angioedema (CREAK), Grenoble Alpes University Hospital (CHUGA), Grenoble, France; 4 Angioedema Center of Reference and Excellence (ACARE), Institute of Allergology, Charité-Universitätsmedizin, Berlin, Germany; 5 Fraunhofer Institute for Translational Medicine and Pharmacology (ITMP), Immunology and Allergology, Berlin, Germany; 6 Department of Vascular Medicine, Amsterdam University Medical Center (UMC), University of Amsterdam, Amsterdam, Netherlands; 7 Department of Immunology, University Hospital Southampton National Health Service (NHS) Foundation Trust, Southampton, United Kingdom; 8 Department of Immunology, Royal Free London National Health Service Foundation Trust, London, United Kingdom; 9 Department of Internal Medicine and Clinical Immunology, National Reference Centre for Angioedema (CREAK), Centre Hospitalier Universitaire de Lille (CHU) Lille, University of Lille, French National Institute for Health Research (INSERM), Institute for Translational Research in Inflammation U1286, Lille, France; 10 Department of Dermatology and Allergology, Helsinki University and Helsinki University Central Hospital, Helsinki, Finland; 11 Operative Unit of Medicine, Angioedema Center, IRCCS Policlinico San Donato, Milan, Italy; 12 Department of Biomedical Sciences for Health, University of Milan, Milan, Italy; 13 Hungarian Angioedema Center of Reference and Excellence, Department of Internal, Medicine and Haematology, Semmelweis University, Budapest, Hungary

**Keywords:** expert consensus, hereditary angioedema, monitoring, prophylaxis, quality of life, shared decision-making, treatment goals, Treat-to-Target

## Abstract

**Background:**

Hereditary angioedema (HAE) is a rare, unpredictable disease that imposes a substantial and multifaceted burden on patients’ daily lives, with many not achieving the World Allergy Organization (WAO)/European Academy of Allergy and Clinical Immunology (EAACI) recommended treatment goal of normalisation of life.

**Methods:**

In March 2025, a European expert committee convened to discuss how to effectively implement WAO/EAACI guidelines in clinical practice, identifying a Treat-to-Target (T2T) approach to support consistent adoption of guideline recommendations into routine care. Through iterative expert discussion and consensus, a T2T algorithm was developed to support the achievement of optimal treatment goals for adolescents and adults with HAE, providing a structured, patient-centred strategy to guide HAE management. Feasibility and clarity of the consensus T2T algorithm were evaluated via an online survey of 64 European clinicians and 15 representatives from patient organisations. Feedback from the survey was used to refine the final version.

**Results:**

The T2T algorithm provides a visual, practical framework that offers guidance on initiating long-term prophylaxis (LTP), as well as setting and assessing individualised short- and long-term targets and treatment plans. Target attainment is achieved through regular monitoring, involving a comprehensive assessment of disease activity and burden, analysis of the causes of breakthrough attacks, and considering optimising management or switching LTP treatment when targets are not being reached. The ultimate goal of treatment for HAE is to achieve normalisation of life.

**Conclusion:**

By setting clear, measurable, and individualised treatment targets supported by shared decision-making, regular monitoring and treatment optimisation as needed, this consensus-derived T2T algorithm aims to promote more consistent care within the HAE community and ultimately improve patient outcomes.

## Introduction

Hereditary angioedema (HAE) is a rare, potentially life-threatening genetic disorder characterised by painful, recurrent, and unpredictable episodes of swelling that most commonly affect the abdomen, extremities, genitals, face, and upper airways (1–4). Without appropriate management and treatment, HAE can lead to considerable disease-related suffering and impaired quality of life (QoL) (5–7). Advances in HAE research over the past decade have resulted in more effective, targeted therapies and updated international, evidence-based management guidelines (8, 9). Despite these advances, challenges remain in the management of patients with HAE, and many patients are still not achieving optimal outcomes (3, 4, 10, 11). Interpretation and implementation of the guidelines can be challenging, leading to inconsistent application of recommended management practices across Europe (4, 12).

The impact of HAE is substantial and multifaceted, encompassing physical, psychological, social, and economic dimensions that extend well beyond the attacks themselves (1, 2, 4, 5, 13, 14). From the patient and caregiver/family perspective, the burden is further compounded when multiple family members are affected (5, 15, 16). Survey data show that nearly half of patients with HAE report missing work or school, and over a third report missing important life events (17). Importantly, disease burden can remain high even when HAE attack frequency is low, with many patients reporting ongoing fear and anxiety between attacks, likely due to the unpredictability of when the next attack might occur (3, 18).

In the management of HAE, treatment strategies include on-demand treatment (ODT) for the acute management of attacks, short-term prophylaxis (STP) to prevent attacks in situations with an increased risk (such as prior to surgical or dental procedures), and long-term prophylaxis (LTP) to prevent attacks and reduce disease burden over time (8).

It is recommended that all attacks should be considered for ODT and treated as early as possible (8). Currently, therapies approved for ODT in Europe include oral sebetralstat as well as injectable icatibant and plasma-derived or recombinant C1 inhibitor (C1INH) therapy (8, 19, 20). While essential for managing HAE attacks, ODT does not prevent attacks (8). Patients who rely solely on ODT may continue to experience an ongoing burden of HAE that impairs QoL (21).

LTP therapies currently approved for first-line use in adults and adolescents with HAE include berotralstat, C1INH therapy, and lanadelumab (8). Most recently, garadacimab and donidalorsen have also been approved for use in this patient population (8, 22–24). The differing routes of administration of LTPs—including oral, intravenous, and subcutaneous formulations—offer patients greater flexibility and choice in tailoring treatment to their preferences and lifestyle needs. Attenuated androgens (AAs) were historically the only option for LTP in HAE. However, their use across Europe has markedly declined following the introduction of targeted LTP therapies and guideline recommendations against the use of AAs (or only as a second-line LTP) due to their limiting side effects and potential for long-term complications (8, 25). Antifibrinolytics, such as tranexamic acid are not recommended for LTP as the data for their efficacy are largely lacking (8). However, antifibrinolytics may benefit some patients and are mainly used when first-line LTP options are unavailable and AAs are contraindicated (8).

According to the 2021 WAO/EAACI guidelines, the ultimate goals of HAE treatment are ambitious – to achieve complete disease control and normalisation of life (8). As stated in the WAO/EAACI guidelines, these treatment goals can only be achieved through LTP, which should be considered for all patients with HAE at every clinic visit and individualised according to clinical need and patient preference (8, 25). However, a practical and actionable approach to support guideline implementation is essential to ensure that HAE treatments translate into meaningful improvements in disease control and normalised QoL.

**Figure 1:**
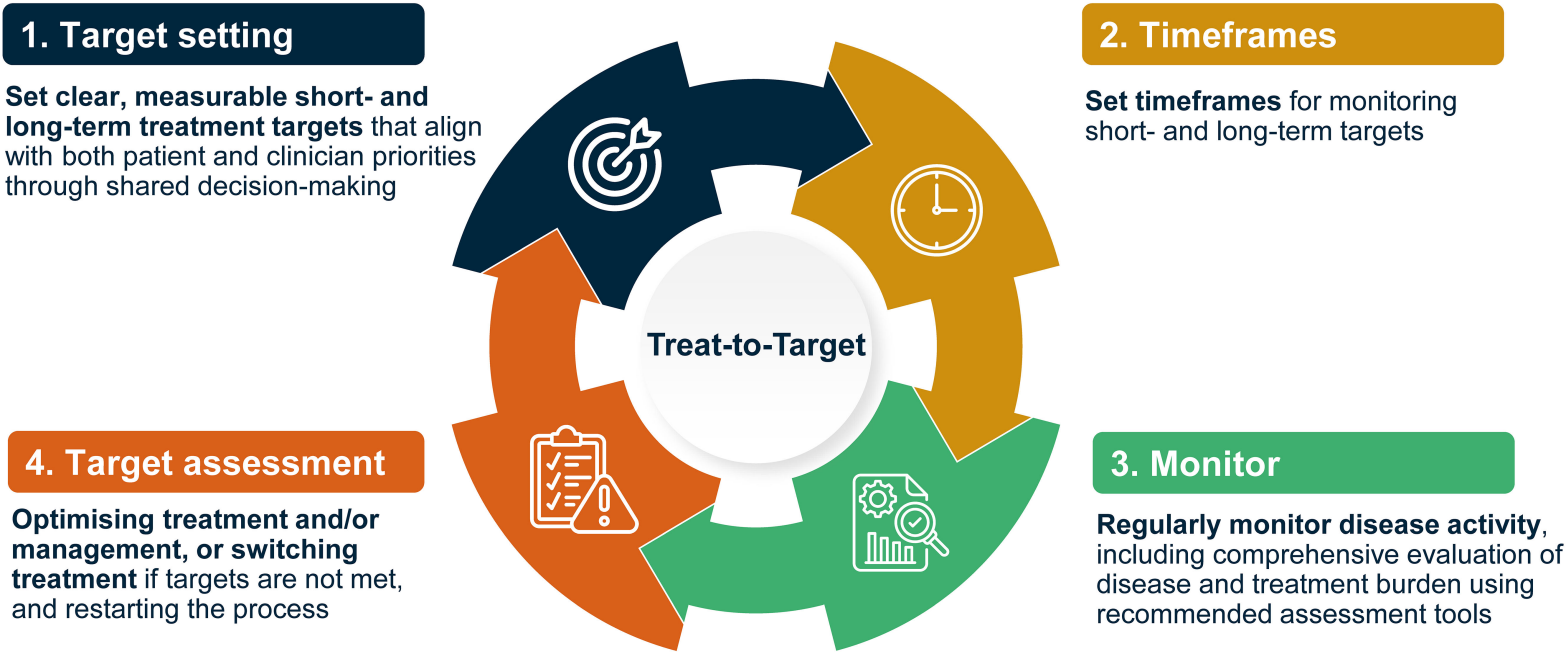
Key principles of a Treat-to-Target strategy (26, 27).

Supporting healthcare professionals in implementing treatment guidelines through a goal-oriented Treat-to-Target (T2T) approach that encompasses shared decision-making and regular, structured monitoring could improve standards of care and consistency in the management of patients with HAE. T2T principles involve setting individualised short- and long-term treatment targets that align with patient and clinical priorities, regularly monitoring progress at set timeframes, and considering optimising management or switching treatment if targets are not reached (**Figure 1**) (26, 27). Initially developed in cardiology, T2T has proven effective in enhancing outcomes across multiple chronic and immune-mediated diseases such as rheumatoid arthritis (RA), psoriatic arthritis and diabetes (26, 28–31).

In March 2025, an expert committee convened to establish consensus on a proposed T2T approach for adolescent and adult patients with HAE, while also seeking input and feedback from the broader clinical community. The outcomes from these discussions are presented here.

## Methods

The committee comprised 11 HAE experts from 8 countries in Europe (Italy, Spain, Hungary, France, Germany, The Netherlands, United Kingdom, and Finland). Across their respective centres, the 11 members of the committee manage over 1,400 patients with HAE. They are actively involved in the national networks of CREAK (French National Reference Center for Angioedema), GSAR (German Society for Angioedema Research), ITACA (Italian network for hereditary and acquired angioedema), SEAIC (Spanish Society of Allergology and Clinical Immunology) and the UK-HAE network. In addition, they practice in Angioedema Centers of Reference and Excellence (ACARE), participate in European Academy of Allergy and Clinical Immunology (EAACI) working groups, and are members of the European Reference Network on Rare Immunodeficiency, Autoinflammatory and Autoimmune Diseases (ERN-RITA).

In March 2025, the committee convened for a two-day, in-person meeting to discuss how to effectively implement international guidelines into clinical practice. To support implementing guidelines and to facilitate setting and the attainment of optimal treatment goals for patients with HAE, the committee, through iterative discussion and expert consensus, collaboratively developed a visual T2T algorithm through consensus as a practical tool for clinical use. The algorithm underwent two rounds of iterative review and refinement via a secure online platform, with revisions informed by group discussion until full committee consensus was achieved.

As part of the development of the T2T algorithm, patient-reported outcome measures (PROMs) or tools were discussed by the committee during iterative group discussions to support target definition and monitoring in clinical practice. The inclusion of specific PROMs (including AECT) was based on expert consensus informed by clinical experience, existing guideline recommendations, and familiarity with their use in routine care.

To refine and validate the feasibility of the proposed T2T approach, feedback and input on the algorithm was gathered from European healthcare professionals currently managing and treating HAE patients and from representatives from HAE patient organisations via an online questionnaire (see **Supplementary Material**). Healthcare professionals were nominated by the committee based on their expertise in treating and managing patients with HAE. Patient representatives were invited from leading HAE patient organisations across Europe. The questionnaires included a combination of closed yes/no questions and open-ended qualitative questions. Healthcare professionals were asked whether they agreed with the proposed T2T algorithm and to provide details of any aspects that should be modified or added, based on their clinical experience of HAE management. The patient organisation questionnaire was designed to obtain feedback on whether they felt that a T2T algorithm for HAE would be a valuable resource in enhancing patient management and optimising treatment outcomes, and whether the short- and long-term targets accurately reflect what patients with HAE are aiming to achieve. Representatives were also invited to propose additions or modifications to the algorithm that they considered relevant to the clinical management of patients with HAE. The comments received from both groups were implemented to refine the final algorithm and confirmed by the expert committee.

## Results

### Overview of T2T algorithm for adolescent and adult patients with HAE

The committee developed a visual algorithm incorporating international guidance, recommendations and shared decision-making principles with the aim of supporting clinical practice and treatment decision-making in HAE. The committee’s findings were further supported by input from 64 European healthcare professionals and 15 representatives from patient organisations, who agreed that the T2T algorithm was a useful tool for clinical practice and supported its publication. The consensus-derived T2T algorithm is the first of its kind developed specifically for HAE.

**Figure 2:**
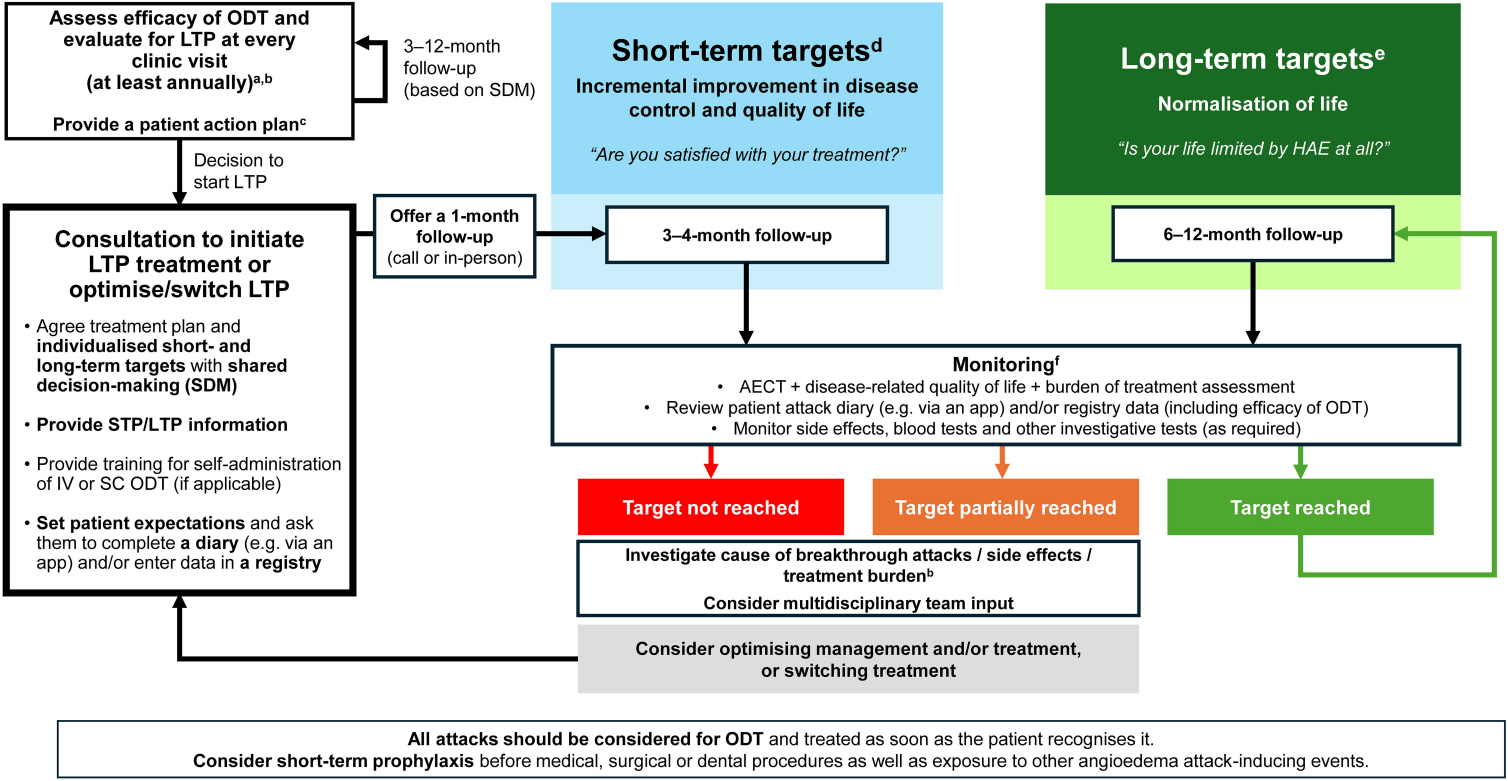
Treat-to-Target algorithm for patients with HAE. **(a)** Evaluate LTP using AECT, disease-related quality of life and burden of treatment assessment, and review of patient attack diary (see point f). **(b)** Attacks/breakthrough attacks can be caused by a variety of factors, such as non-adherence, comorbidity, infection, lifestyle changes (such as stress), ACE inhibitors, oestrogen, puberty and pregnancy. It is important to identify triggers before deciding whether to optimise and/or switch treatment. **(c)** Action plan should include emergency contacts, acute management instructions, and STP information. Provide training for self-administration of IV or SC on-demand therapy (if applicable). **(d)** When setting short-term targets, consider: A meaningful reduction in attacks, improved ability to carry out daily activities, and treatment satisfaction and tolerance. Consider a treatment transition period to allow treatment to reach maximum efficacy and to account for any washout or overlap when switching treatments. **(e)** When setting long-term targets, consider: Achievement of patients’ potential, living without fear or limitations to daily life, being attack-free, and experiencing minimal treatment burden. **(f)** Monitoring and assessment: There are multiple factors, on top of AECT, to consider when assessing response to treatment including severity and location of attacks, quality of life domains (psychological burden, impact on work/education, impact on social/leisure activities, lifestyle), efficacy of ODT (frequency of ODT use and need for >1 ODT injection per attack), occurrence of prodromal symptoms, treatment satisfaction, side effects, adherence to treatment, access to medication and capability to administer treatment. At a minimum, the AECT should be conducted on each clinic visit to provide an objective baseline. Additional assessment tools may also be helpful, or a tailored approach using appropriate questions focussed on the areas above, depending on the patient’s individual needs. ACE, angiotensin converting enzyme; AECT, Angioedema Control Test; LTP, long-term prophylaxis; ODT, on-demand treatment; SDM, shared decision-making; STP, short-term prophylaxis.

The algorithm (**Figure 2**) provides a structured, step-by-step approach that includes recommendations for regular assessment of patients on ODT only or LTP, establishing treatment plans and individualised short- and long-term targets through shared decision-making, guidance for initiating or optimising/switching LTP, regular monitoring for target achievement (including the use of Angioedema Control Test [AECT] plus comprehensive assessment of disease-related QoL and burden of treatment – refer to ‘Monitoring of treatment targets’ section), and optimising management or switching treatment if targets are not being met or are only partially met. Additional details on each step are provided in the algorithm appendix (**Figure 2** **caption**). The individual components of the algorithm are discussed below.

### Evaluation for LTP at every clinic visit

Given the unpredictability of HAE attacks, all patients should have access to ODT for treatment of HAE attacks, as recommended by the guidelines (8). Newly diagnosed patients should be provided with a patient action plan that includes contact information for emergency support, instructions for ODT (including training on how to self-administer ODT if applicable), and information on STP and LTP (8).

For patients managed exclusively with ODT, a 3-month follow-up is recommended after the first appointment or diagnosis and then every 9–12 months. Earlier reassessment may be warranted in cases of increased attack frequency or severity or based on individualised patient considerations.

Current international guidelines recommend assessing the need for LTP at every clinic visit (8); however, thresholds for initiating LTP (usually based on HAE attack rate) vary according to national regulations. Multiple factors should be considered via as shared decision-making process when evaluating the need for LTP, including the frequency, severity, and location of attacks; patient preferences and burden of current treatment; the impact of HAE on QoL—encompassing psychological burden, work or education, personal and family life, and lifestyle considerations (including social/leisure activities); access to and ease of administration of ODT; as well as side effects and adherence to treatment (8, 32).

Before initiating LTP, a comprehensive assessment of disease control (using AECT) and disease activity (e.g. review of patient attack diaries or registry data; validated questionnaires such as Angioedema Activity Score [AAS] or Hereditary Angioedema Activity Score [HAE-AS]) should be performed, alongside evaluation of disease-related QoL and burden of disease (see ‘Monitoring of treatment targets’ section). These assessments can serve as a baseline reference for assessing treatment effectiveness.

When discussing LTP options and treatment targets through shared decision-making, patients may struggle to evaluate choices or have differing expectations if they do not have clear information about the benefits and risks of their available options (32). Patients should be educated about the central role they play in decision-making and be provided with effective tools (e.g. digital decision aids, infographics, leaflets) to help them understand their options (i.e. oral or injectable treatment) and implications of their choices. Emotional support is essential to enable patients to articulate their values and preferences and to ask questions freely, without worry of disapproval from their clinician (33). Some patients may feel more comfortable discussing concerns with nurses (34), although dedicated nursing support is not available in all clinics or countries. Shared decision-making is consistently emphasised in guidelines as best practice and is embedded into the T2T framework (8).

### Short- and long-term targets

To accomplish optimal treatment outcomes, individualised short- and long-term targets should be set to ensure that the patient’s priorities and needs are fully captured. Follow-up intervals for assessing short- and long-term targets were defined by expert consensus informed by clinical experience and typical practice.

#### Assessing short-term targets

**Figure 3:**
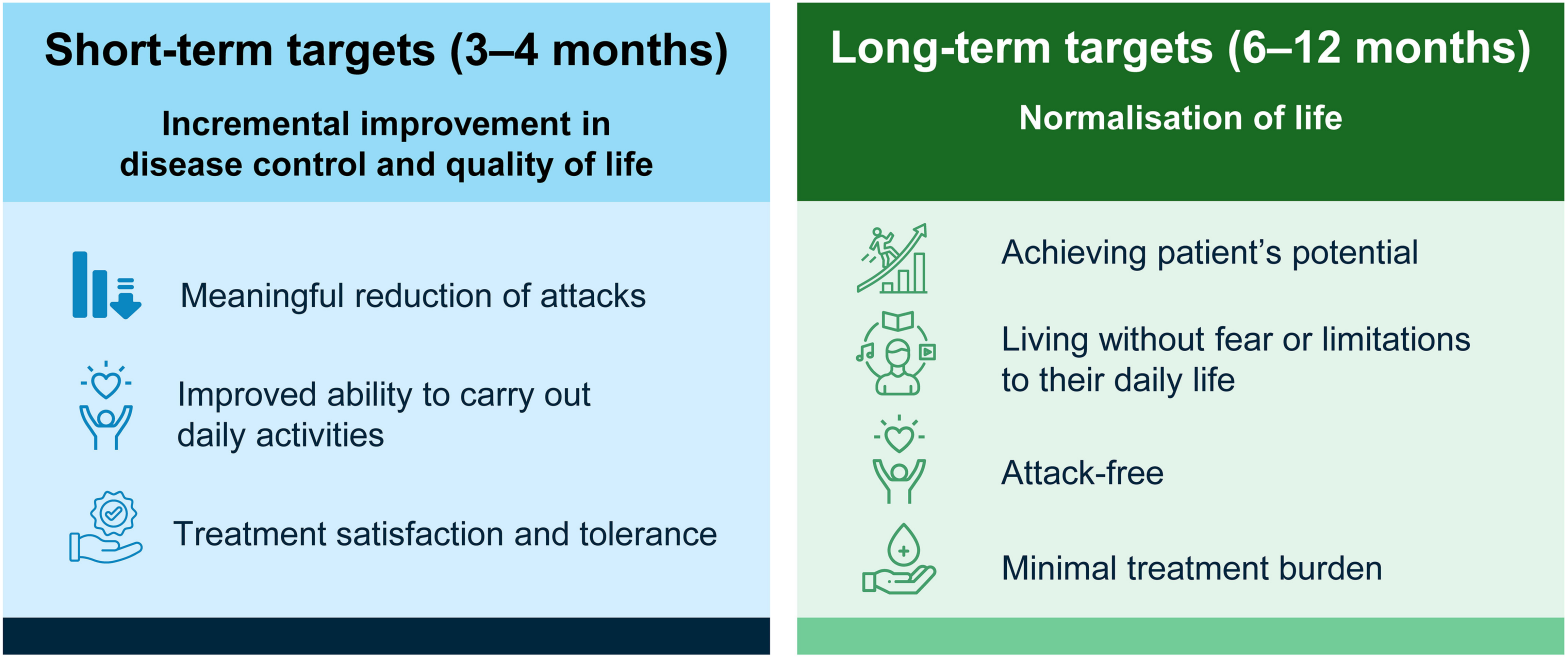
Short- and long-term targets in a Treat-to-Target approach.

Incorporating short-term targets is important in HAE, as a treatment transition period is typically required to allow treatment to achieve maximal effect and for any potential side effects to be managed and resolved. Short-term targets should be assessed after 3–4 months, aiming for an incremental improvement in disease control and QoL (**Figure 3**). When setting short-term targets, consider a meaningful reduction in attacks (within the context of the patient’s baseline number of attacks), improved ability to carry out daily activities, and treatment satisfaction and tolerance.

While no definition of incremental improvement in disease control and QoL has been formally established, the minimum clinically important difference (MCID) for AECT has been determined as three points for improvement in angioedema control and can provide an objective measure of disease control alongside trends seen in a patient’s attack diary and levels of patient satisfaction and treatment burden (35).

#### Assessing long-term targets

**Figure 4:**
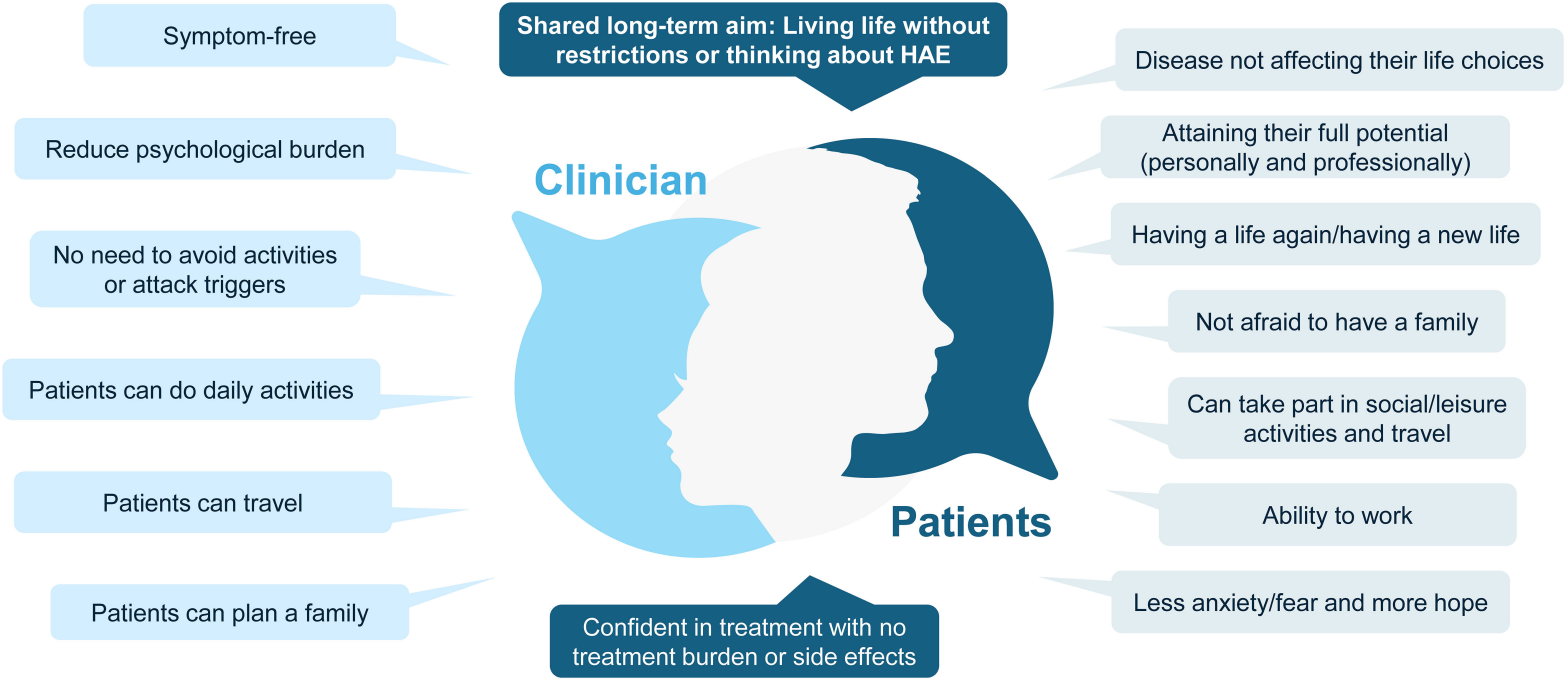
Clinician and patient definitions of ‘normalisation of life’. Definitions shown are the opinions from the European Expert Committee.

Assessed after 6–12 months, long-term targets should aim for the ultimate goal of normalisation of life (**Figure 3**). This not only includes being attack-free but also achieving a patient’s potential—experiencing minimal treatment and psychological burden and living without fear or limitations to daily life or activities. It is important to understand what normalisation of life means for each individual patient and setting these targets should rely on a shared decision-making process. Perceptions of ‘normal life’ may also change over time and should be reviewed at each follow-up appointment (**Figure 4**).

### Setting patient expectations when initiating or optimising/switching LTP

When initiating or switching LTP treatment, it is important to set patient expectations for the treatment prescribed; to provide adequate education on potential side effects and the importance of allowing treatment to reach maximum efficacy, and to account for any washout or overlap when switching treatments. Treatment adherence can be poor if patients do not have all this key information beforehand (36, 37).

Patients should be educated on how to self-manage potential side effects and advised to contact their HAE physician if any issues arise. Many HAE treatments have expected side effects, and experience has shown that patients who are well educated about these side effects are more likely to be satisfied with and adhere to their treatment (38). It is also important to inform patients that interventions are available to help manage side effects (e.g. adjuvant treatment to help relieve gastrointestinal symptoms, appropriate training in injection techniques to minimise injection-site reactions), and encourage them to discuss any need for such support (38). Patients should also be informed of and psychologically prepared for the possibility of attacks upon initiating or switching LTP and should have access to ODT to treat any such attacks (see ‘Switching LTP’ section).

### Use of patient diaries and/or registries for recording HAE attacks

All patients should keep a patient diary (e.g. paper, electronic or via an app such as HAE TrackR) to record the details of each HAE attack or to submit this information through a registry. The information captured should include the date and time of the attack, affected body area, severity (0–4 representing very mild to very severe), triggers (if known), warning signs (if any), ODT used (including dose, date, and time of administration) and the duration of the attack.

### One-month follow-up after LTP initiation

The experts agreed that offering patients a follow-up visit or check-in call one month after initiating LTP is valuable for assessing early treatment experience and providing reassurance, if needed. Patients may feel uncertain with a new treatment approach, or after a switch of LTP, which can lead to unnecessary discontinuations. If side effects are reported or attacks persist, patients can be counselled that adverse effects may resolve over time, that certain interventions and self-administration training may help manage side effects, and that maximum therapeutic efficacy may require a longer treatment duration.

### Monitoring treatment targets

Treatment targets should be monitored at every clinic visit using the AECT, together with assessment of disease-related QoL, burden of treatment, and regular review of the patient diary. Because treatment targets should be individualised, they need to be assessed on a case-by-case basis and incorporated into the shared decision-making process. Reliable assessment of target achievement requires longitudinal use of the same PROMs/tools within each patient.

A comprehensive assessment is required, on top of AECT, when assessing response to treatment including severity and location of attacks, QoL domains (psychological burden, impact on work/education, impact on social or leisure activities, lifestyle), treatment satisfaction (including being able to self-manage HAE), treatment burden (including side effects, adherence, access to medication, and ability to self-administer treatment), efficacy of ODT (frequency of ODT use and need for more than one ODT injection per attack), and occurrence of prodromal symptoms. At a minimum, the AECT should be conducted at each clinic visit to provide an objective baseline. It is also important to ask patients open-ended questions at each assessment and to actively listen to understand their satisfaction with their treatment and whether they feel they are achieving their individual goals.

Additional assessment tools to assess disease activity, QoL and treatment satisfaction (e.g. AAS, HAE-AS, Angioedema Quality of Life questionnaire [AE-QoL], Hereditary Angioedema Quality of Life questionnaire [HAE-QoL] and Treatment Satisfaction Questionnaire for Medication [TSQM]) may also be helpful. Alternatively, a tailored approach using appropriate questions focussed on the areas above may be considered, depending on the patient’s individual needs. However, the experts agreed that there is currently an unmet need for an HAE-specific tool that is simple to use in clinical practice and comprehensively monitors all aspects of the disease, including satisfaction with the treatment.

Regular monitoring should continue typically every 12 months with well-controlled disease and more frequent for uncontrolled disease. The follow-up timelines are considered best practice and are guidance only, based on expert opinion and experience (8).

### Assessing target achievement

If targets are not achieved or only partially met, it is important to consider the causes of breakthrough attacks, side effects, and treatment burden. Also, clinicians should establish whether symptoms are attributed to HAE or to another condition with similar symptoms. Breakthrough attacks can be triggered by a variety of factors, such as non-adherence, comorbidity, infection, lifestyle changes (such as stress), angiotensin converting enzyme (ACE) inhibitors or other concomitant therapies, oestrogen, puberty and pregnancy (8, 39–41). Within a T2T strategy, clinicians should systematically identify potential triggers of breakthrough attacks and manage them with support from the multidisciplinary team.

After excluding non-HAE causes for not achieving targets, consider optimising management and/or treatment, or consider switching treatment, if appropriate.

Poor adherence is a common reason for not achieving treatment targets and should also be explored with patients during target assessment (42–45). Wording questions about adherence in terms such as, “How often do you miss your HAE medication?” helps normalise the topic and can make patients feel more comfortable discussing adherence challenges (46).

For some patients, treatment targets may only be partially achieved. For example, a patient may be attack-free yet experience a high treatment burden, which could be addressed by increasing the dosing interval or by switching to an alternative LTP (47). Furthermore, a patient’s QoL may remain impaired despite a low attack rate, prompting consideration of treatment optimisation or switching LTP.

### Multidisciplinary team input

Optimal management of HAE extends beyond pharmacological therapy, requiring highly specialised and multidisciplinary-led care for optimising treatment and outcomes. Healthcare professionals should refer patients with suspected HAE to specialist centres so that patients are treated by a specialist with expertise in managing HAE (8). Depending on the patient’s needs, consider, for example, consultation and coordination with gynaecologists (for contraceptive advice), obstetricians (in pregnant patients), psychologists (for psychological burden), geneticists/genetic counsellors, respiratory or ear, nose, and throat specialists, gastroenterologists, emergency medicine physicians, and other specialists managing comorbidities.

### Switching LTP

The protocol for switching will depend on the reasons for the switch and on the LTP switched from and to. The decision to switch therapy may be determined by multiple factors, including side effects, patients failing to achieve a meaningful reduction in attacks, patient preference, treatment burden, or impact on QoL (refer to ‘Short- and long-term targets’ section) but should always be made on a case-by-case basis.

Prior to a treatment switch, it is important to set realistic treatment expectations with the patient and inform them of the onset time, side effects, and potential overlap strategies. Switching or optimising therapy must be a shared decision with the patient and may be limited by available treatment options.

## Discussion

Therapeutic advances have transformed the HAE management landscape, offering patients the potential to achieve complete disease control and normalisation of daily life (8). Implementing a T2T approach to HAE management—setting individualised, measurable short- and long-term targets—provides a framework for optimising patient outcomes.

The T2T approach was first introduced within the field of cardiology and has since been shown to improve outcomes for patients versus routine practice in other chronic, immune-mediated, or progressive conditions, including hypertension, psoriatic arthritis, rheumatoid arthritis, systemic lupus erythematosus, atopic dermatitis, inflammatory bowel disease, asthma, diabetes, and multiple sclerosis (26–31, 48–56). The pivotal Systolic Blood Pressure Intervention Trial (SPRINT) provided early and influential evidence for the benefits of a T2T approach, demonstrating that treating systolic blood pressure to a target of less than 120 mmHg significantly reduces rates of cardiovascular events and death (53). In rheumatology, real-world application of a T2T strategy in patients with psoriatic arthritis, rheumatoid arthritis, and systemic lupus erythematosus has been shown to result in more patients achieving and sustaining remission targets and reduced disease activity (28, 29, 54–56).

Shared decision-making is fundamental to the success of a T2T approach, being an ongoing, iterative process that adapts to the patient’s evolving needs and response to therapy (32). Routine engagement of patients in discussions about treatment options, expected benefits, potential risks, and practical considerations is essential for effective HAE management. Incorporating patient preferences and disease impact into clinical decision-making improves adherence, supports sustained disease control, and ensures that target-oriented strategies remain both realistic and meaningful for the individual (32).

The PROMs or tools referenced in the algorithm are intended as illustrative, non-exhaustive examples of validated instruments that may be used to support a T2T approach, rather than as a list of mandatory tools. This illustrative approach reflects the committee’s recognition of an unmet need for a simple, HAE-specific tool suitable for routine clinical use that comprehensively captures all relevant aspects of disease burden.

Although this is the first T2T algorithm developed specifically for HAE, a Spanish Delphi expert consensus published in 2023 previously discussed the possibility of a T2T management approach in HAE and emphasised the importance of clear treatment goals and individualised, shared decision-making (57). The T2T algorithm presented here builds upon current WAO/EAACI guidelines and consensus reports and is based on the opinion and experience of 11 HAE experts from across Europe, together with input from 64 additional European healthcare professionals who manage and treat patients with HAE and 15 representatives from leading HAE patient organisations. While this broad, multidisciplinary input strengthens the relevance of the recommendations, a limitation of this work is that they were developed through expert consensus based on iterative discussion rather than a formal, structured consensus methodology, such as a scale-based or Delphi process with predefined agreement thresholds or quantitative analysis.

The strong consensus among experts regarding persistent unmet needs and challenges in HAE highlights the need for a more standardised approach to care and practical support for guideline implementation.

The committee recognises that additional research is needed to help support the implementation of this approach, which includes:

- Consensus on clearer and tangible definitions of ‘normalisation of life’
- Development of additional simple and clinically feasible HAE-specific PROMs or tools to capture all aspects of HAE burden (treatment burden and impact on QoL) to enable comprehensive assessment and support shared decision-making
- Quantitative definitions on how treatment targets are reached
- Use of a formal consensus methodology, such as a Delphi-based or scale-driven approach, to further validate, prioritise, and operationalise the proposed T2T framework, including the selection and weighting of relevant HAE-specific PROMs or tools
- Development of tailored management approaches for special populations (e.g. paediatrics, pregnant individuals, and the elderly)
- Prospective studies applying the T2T algorithm in routine clinical practice to evaluate its impact on clinical outcomes and patient-reported measures

Implementing a T2T approach in the management of HAE may support healthcare professionals in achieving optimal patient outcomes—ultimately aiming for normalisation of life—by providing a pragmatic, evidence-informed, structured approach to applying international guideline recommendations in clinical practice. As an expert consensus-based framework, the proposed T2T approach is intended to evolve as new evidence emerges, and prospective clinical validation will be important to assess and refine its clinical utility.

## Data Availability

The original contributions presented in the study are included in the article/**Supplementary Material**. Further inquiries can be directed to the corresponding author.
