# Supplementary material for: A Treat-to-Target approach in hereditary angioedema: expert consensus from a European committee
Source: Front Immunol. 2026 Feb 12;17:1773279. doi: 10.3389/fimmu.2026.1773279 (PMC12936744; doi:10.3389/fimmu.2026.1773279)
Supplement: Supplementary file 1 [file Table1.docx]

Supplementary Material

| We value your expertise and invite your feedback to help finalise this Treat-to-Target algorithm for the HAE community.   1. Do you agree with the proposed Treat-to-Target algorithm for HAE developed by the HAE3 European Steering Committee?    1. Yes    2. Yes – but with points clarified in the body of the manuscript (please answer Question 2)    3. No   If no, please provide your reason(s) why below, and if possible, solutions/proposed changes. Please provide your contact details in Question 3 if you are happy for us to contact you regarding your comment.  _______________________________________________________________________   1. Are there any aspects of the algorithm you would like clarified further in the final manuscript?   _______________________________________________________________________   1. If you would like to be listed as a contributor on the manuscript, please provide your details below    1. Title    2. First name    3. Middle initials    4. Surname    5. Affiliation    6. Country    7. Email address |
| --- |

**Supplementary Figure 1.** Online questionnaire distributed to European HCPs who manage and treat patients with HAE.

| We value your expertise and support in the HAE community and invite your feedback to help finalise this Treat-to-Target algorithm for HAE.   1. Would you agree that publishing the proposed Treat-to-Target algorithm for HAE will be a valuable resource in enhancing patient management and optimising treatment outcomes for those living with HAE?    1. Yes    2. No   If no, please provide your reason(s) why below, and if possible, solutions/proposed changes. Please provide your contact details in Question 4 if you are happy for us to contact you regarding your comment.  _______________________________________________________________________   1. Do you feel that the short-term and long-term targets outlined in the algorithm and appendix accurately reflect what patients with HAE are aiming to achieve?    1. Yes    2. Yes, but with points clarified in the body of the manuscript    3. No   If you selected ‘Yes, but with points clarified in the body of the manuscript’ or ‘No,’ please specify which aspects require further clarification or the reasons why. If possible, please also suggest solutions or proposed changes. Please provide your contact details in Question 4 if you are happy for us to contact you regarding your comment.  _______________________________________________________________________   1. Are there any other aspects of the algorithm you would like clarified further in the final manuscript?   _______________________________________________________________________   1. If you would like to be listed as a contributor on the manuscript, please provide your details below    1. Title    2. First name    3. Middle initials    4. Surname    5. Patient organisation    6. Country    7. Email address |
| --- |

**Supplementary Figure 2.** Online questionnaire distributed to patient representatives from leading HAE patient organisations across Europe.
